# Socioeconomic Inequalities Worsen the Risk of Death in CKD: A Population-Based Cohort Study in Italy

**DOI:** 10.34067/KID.0000000592

**Published:** 2024-09-26

**Authors:** Marta Giaccari, Claudia Marino, Pietro Manuel Ferraro, Giulia Cesaroni, Marina Davoli, Nera Agabiti

**Affiliations:** 1 PhD Program in Experimental and Translational Medicine, Università Cattolica del Sacro Cuore, Rome, Italy; 2 Department of Epidemiology of the Regional Health Service, ASL Roma 1, Rome, Italy; 3 Section of Nephrology, Department of Medicine, Università degli Studi di Verona, Verona, Italy

**Keywords:** CKD, chronic kidney failure, chronic renal disease, chronic renal failure, chronic renal insufficiency, clinical epidemiology, epidemiology and outcomes

## Abstract

**Key Points:**

- Socioeconomic position is associated with mortality in patients with CKD.
- Socioeconomic inequalities are stronger in men then in women.
- There was no evidence of association between deprivation and ESKD.

**Background:**

Few studies have explored the effect of socioeconomic position on CKD. This study aims to fill this gap using a large Italian cohort of patients with CKD.

**Methods:**

We analyzed a cohort of incident CKD cases from the Lazio Regional Health Information System from January 1, 2012, to December 31, 2021. We used the deprivation index (DI), a five-category census-block indicator that integrates several dimensions of disadvantage. The outcomes were mortality and ESKD. We characterized the health status of patients in the 2 years before CKD identification and followed each participant from the index date to the end of follow-up (*i.e*., the date of the outcome, of emigration, or December 31, 2022, whichever came first). We used Cox proportional hazard models to investigate the association between DI and outcomes (hazard ratio, 95% confidence interval [CI]).

**Results:**

From 2012 to 2021, 127,457 new cases (55.9% men) were diagnosed. The average age was 72.2 (±13.7) for men and 74.4 (±14.8) for women. During an average follow-up of 4.3 years (±3.2), 57,158 patients died (45%), and 5994 developed ESKD (5%). The age-adjusted association between DI and mortality was higher in men than in women (*P* value interaction = 0.02), hazard ratios for the extreme categories of DI (very high versus very low) were 1.16, 95% CI, 1.12 to 1.21 for men, and 1.08, 95% CI, 1.04 to 1.13, for women. There was no evidence of association between DI and ESKD.

**Conclusions:**

In this population, socioeconomic disadvantage is associated with a higher risk of death, but not of ESKD, in patients with CKD.

## Visual Abstract

**Figure:**
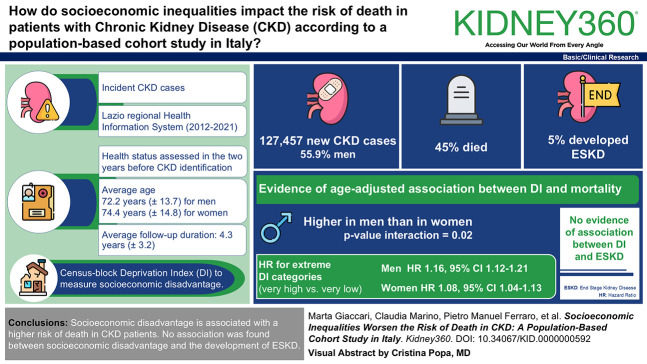

## Introduction

CKD is a pathologic condition of kidney damage sustained over at least 90 days, defined by a low GFR, commonly estimated from serum creatinine and high urine albumin-creatinine ratio.^1^ However, because it is generally asymptomatic, patients often are referred to nephrologists very late, when dialysis is the only remaining option. According to the IQVIA Longitudinal Patient Database in Italy, 66,676 patients were defined as incident CKD cases with eGFR values ≥30 and ≤60 ml/min per 1.73 m^2^ in two consecutive records that were 90 days apart. The study illustrated that after 6 months from the incident CKD, 77% of patients remained undiagnosed.^2^ This phenomenon causes high costs for health care systems.^3^ In 2017, the global prevalence of CKD was 9.1%, with most of the burden affecting countries with a low sociodemographic index.^4^ Use of KRTs is widespread but costly. It is projected to involve more than 5 million people globally by 2030.^5^ Furthermore, many patients with CKD die prematurely because CKD is undiagnosed or because KRTs are not accessible.^5^ Therefore, it is imperative to develop strategies to accurately identify cases at risk of CKD and estimate the real burden of the disease.

Digital health information systems (HIS) contain large amounts of data, allowing researchers to make comparisons across different countries. Recently, Sundström *et al.* published data assembled from the HIS of 11 countries.^6^ The authors evaluated various definitions of CKD, based on laboratory data and HIS diagnostic coding. They found that possible CKD cases were present in one out of ten patients. Notably, annual death rates were high both in the group of patients identified by laboratory values and by diagnostic code (6% and 9%, respectively).^6^

Socioeconomic position (SEP) is a strong predictor of increased risk for many chronic conditions,^7,8^ particularly CKD.^9^ Thus, the screening for these pathologies is essential in the most disadvantaged populations. When Ghazi *et al.* added neighborhood SEP to the screening for CKD, the sensitivity was amplified at the cost of decreased specificity.^10^ Low SEP has also been associated with all-cause mortality in patients with incident CKD,^11–13^ although this association was not confirmed in an English cohort.^14^ The association between SEP and progression to ESKD is even more controversial.^11,14,15^ Previous studies have been limited, mainly by short follow-up periods and small-scale cohorts. Moreover, the indicators used to assess SEP differ across studies and have different meanings, making interpreting and comparing the results very challenging. In Italy, more evidence needs to be produced regarding this theme. Nonetheless, if data on the progression to ESKD and mortality were confirmed, SEP could be an essential aide to CKD risk stratification to identify patients at risk of poor outcomes (such as ESKD and mortality) who may benefit from additional services.

We hypothesized that disadvantaged SEP could directly affect the outcomes associated with CKD. Therefore, the aim of this study was to investigate this possible association among incident CKD cases in patients in the Lazio region, focusing on the two main outcomes: mortality and ESKD requiring KRTs.

## Methods

### Study Design

This study is a cohort study based on HIS data.

### Setting

With its 5.5 million inhabitants (9.7% of the whole country), the Lazio region is the central region of Italy containing the capital (Rome). The population in this region is slightly younger than Italy as a whole, with the percentage of people over 65 at 21.7% in the former versus 22.8% in the latter.^16^ In 2017, the standardized prevalence rate of CKD was 1.76%, 2.06% for male patients and 1.50% for female patients. The prevalence increased with age, rising from 0.33% (age 0–18) up to 14.18% (age older than 85) among male patients and from 0.25% up to 8.18% among female patients.^17^

### Data Sources

We used the Lazio HIS, in particular, the Regional Health Assistance Patients Registry, Hospital Discharge Registry, Outpatient Specialist Service Information System, Copayment Exemption Registry and Drug Dispensing Registry, the Regional Register of Causes of Death, and the Lazio Dialysis Registry, which all have a good level of quality and completeness.^18,19^ All residents of the Lazio region served by the public health service have a personal identification number recorded in all the regional health care databases. This individual identifier provides the key to linking all regional databases and allows the unique identification of individuals within the regional health system.

### Study Population

We identified patients with an incident CKD diagnosis between January 1, 2012, and December 31, 2021, using a validated algorithm.^17,20^ In brief, we selected patients who had received a copayment exemption, were discharged for CKD, or had nephology visits with appropriate drug prescriptions or urine albumin measurements. The codes for the selection are listed in Supplemental Table 1, and more details on the algorithm can be found in Marino *et al.*^17^ The index date was defined as the first time a patient was recorded in the HIS due to CKD as defined above. To determine what to include in the study population, we only checked incident cases in the 2 years before the index date for CKD diagnosis and excluded prevalent cases. Because we selected the first CKD case during the period 2012–2021, this check was necessary only for patients who were identified in 2012 and 2013. We excluded patients younger than 18 years and those who had undergone KRTs at the index date (for codes see Supplemental Table 1).

### Follow-Up

The follow-up period started at the index date. It ended at the date of death, of KRTs, of censoring (migration from the Lazio region), or December 31, 2022 (the end of the study period), whichever came first.

### Outcomes

The end points were all-cause mortality and ESKD requiring KRTs (Supplemental Table 2). For convenience, from this point on, we will refer to the latter simply as ESKD.

### Exposure

We used the Italian deprivation index (DI) as a measure of SEP.^21^ The DI was attributed to the census block of residence at the index date and included the following parameters: level of education, unemployment, home ownership, single-parent families, and overcrowding. The DI divides the Lazio census blocks into five classes according to the quintiles of the population distribution (where very low DI represents the least deprivation and very high DI the most deprivation).

### Other Variables

We considered age a potential confounder in the association between DI and the study outcomes and sex a potential effect modifier.

We used discharge diagnoses from the 2 years preceding the index date as a proxy for patients' comorbidities. Binary variables (yes versus no) were evaluated for the following conditions: cancer, diabetes, obesity, lipid metabolism disorders, anemia, dementia, hypertension, ischemic cardiopathy, arrhythmias, heart failure, cerebrovascular diseases, peripheral vascular diseases, chronic obstructive pulmonary disease, and liver diseases. We identified these conditions using the codes listed in Supplemental Table 3.

Because individuals in different DI classes have a heterogenous geographical distribution, which might affect the accessibility of care and KRTs, we considered the residential area, categorized as Rome Municipality, Rome Province, and other Lazio Municipalities, representative of rural areas, as an additional confounder.

### Statistical Analyses

We presented sociodemographic characteristics and comorbidities as percentages or mean and SD across DI categories. We tested the relationship between categorical and continuous variables with DI using the chi-squared test or ANOVA, respectively. We calculated cumulative probabilities and crude mortality rates (CMRs) with 95% confidence interval (CI) per 100 person-years by DI.

We used Cox proportional hazard models to estimate the association between DI and the outcomes, producing hazard ratios (HRs) with 95% CI and using very low DI as the reference category. We checked the proportional hazard assumption both by graphical inspection and formal test (Schoenfeld Residuals Test).

We tested for interaction between DI and sex using the Wald test. Because we found a statistically significant interaction between sex and DI for the mortality outcome, we conducted the analysis separately for men and women. We developed different statistical models. In the first one, we evaluated the crude association between DI and the outcomes; in the second one, we also adjusted for age. In the third model, we introduced other variables: residence and comorbidities (cancer, diabetes, obesity, lipid metabolism disorders, anemia, dementia, hypertension, ischemic cardiopathy, arrhythmias, heart failure, cerebrovascular diseases, peripheral vascular diseases, chronic obstructive pulmonary disease, and liver diseases) to analyze the possible role of the most common clinical risk factors on the studied associations using a stepwise approach. We considered the age-adjusted model as the main model.

In studying ESKD, we hypothesized that highly deprived individuals could have died before the occurrence of ESKD. Thus, we used Cox models to analyze the association between DI and ESKD, considering death as a competing risk for ESKD. We performed all the analyses using SAS version 9.4 (SAS Institute, Cary, NC).

Ethics committee approval for the study was not necessary because the data used were already collected. The data were analyzed anonymously through a standardized methodology according to the Italian national privacy law and the Declaration of Helsinki. The Department of Epidemiology of the Lazio Regional Health Service is the regional referral center for epidemiological research, and it has full access to anonymized HIS.

## Results

**Figure 1:**
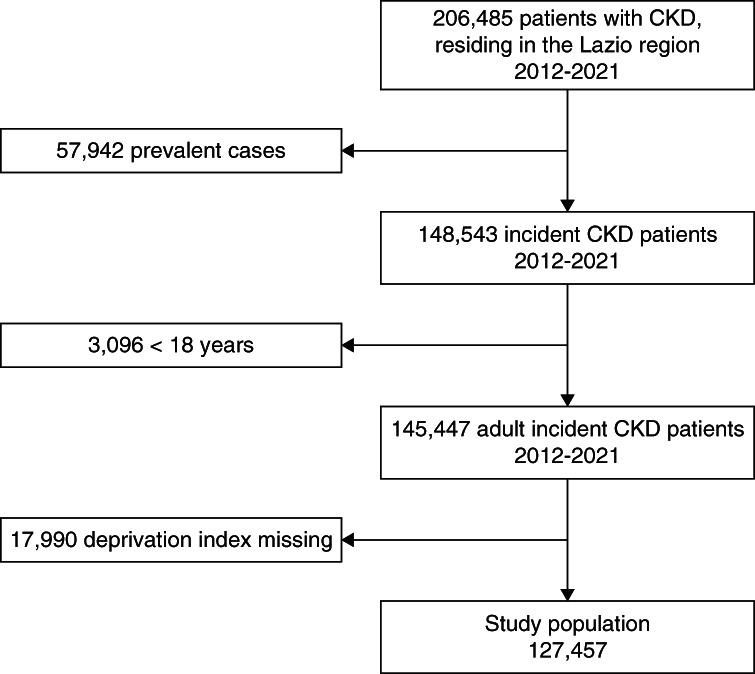
Flow chart of the selection of the study population.

We identified 206,485 patients residing in the Lazio region with CKD. Of these, 57,942 were prevalent cases and therefore excluded. We also excluded 3096 children and 17,990 patients without DI information. Overall, we included 127,457 patients in the study (Figure 1). During the study period, 4210 (3%) patients emigrated from the Lazio region and were lost to follow-up; 61,097 (48%) patients were followed until December 31, 2022, while 62,150 (49%) ended the follow-up because of the occurrence of study outcomes.

Table 1 shows the baseline characteristics of the study population. Patients were less represented in the very low DI class (14%). The rest were equally distributed across the other DI classes. The average age was 73 years (SD, 14), and the mean age decreased with increasing DI (*P* value < 0.001). In proportion, most patients were men (chi-squared *P* value < 0.001). However, the proportion of women was higher in the most deprived classes (chi-squared *P* value < 0.001). Most comorbidities were unevenly distributed across the DI categories, with higher prevalence in the most disadvantaged participants. For example, the prevalence of diabetes was 15% in the very low DI versus 18% in the very high DI chi-squared *P* value < 0.001.

**Table 1:** Clinical and sociodemographic characteristics of the study population

Characteristics	Total		DI															*P* Value
			Very Low			Low			Medium			High			Very High			
	*N*	Col %	*N*	Col %	Row %	*N*	Col %	Row %	*N*	Col %	Row %	*N*	Col %	Row %	*N*	Col %	Row %	
All	127,457	100	17,359	100	14	27,087	100	21	27,799	100	22	28,437	100	22	26,775	100	21	
**Sex**																		<0.001
Men	71,187	56	10,076	58	14	15,150	56	21	15,480	56	22	15,891	56	22	14,590	54	20	
Women	56,270	44	7283	42	13	11,937	44	21	12,319	44	22	12,546	44	22	12,185	46	22	
Age, mean (SD)	73.1 (14.2)		75.0 (13.7)			74.2 (13.9)			72.8 (14.4)			72.3 (14.4)			72.2 (14.3)			<0.001
**Age (classes)**																		<0.001
Younger than 65	28,878	23	3193	18	11	5596	21	19	6573	24	23	6969	25	24	6547	24	23	
65 years or older	98,579	77	14,166	82	14	21,491	79	22	21,226	76	22	21,468	75	22	20,228	76	21	
**Residence**																		<0.001
Rome municipality	63,360	50	12,185	70	19	16,555	61	26	11,505	41	18	9708	34	15	13,407	50	21	
Rome province	27,684	22	1496	9	5	3500	13	13	7240	26	26	9274	33	33	6174	23	22	
Other Lazio's municipalities	36,413	29	3678	21	10	7032	26	19	9054	33	25	9455	33	26	7194	27	20	
**Comorbidities**																		
Hypertension	35,959	28	4971	29	14	7596	28	21	7674	28	21	7861	28	22	7857	29	22	<0.001
Arrhythmias	22,544	18	3250	19	14	4915	18	22	4745	17	21	4961	17	22	4673	17	21	<0.001
Diabetes	20,532	16	2554	15	12	4006	15	20	4329	16	21	4723	17	23	4920	18	24	<0.001
Heart failure	20,328	16	2711	16	13	4322	16	21	4272	15	21	4551	16	22	4472	17	22	<0.001
Ischemic cardiopathy	18,877	15	2614	15	14	4015	15	21	3888	14	21	4173	15	22	4187	16	22	<0.001
Cancer	14,535	11	2190	13	15	3218	12	22	3080	11	21	3060	11	21	2987	11	21	<0.001
Cerebrovascular diseases	14,436	11	2030	12	14	3063	11	21	3058	11	21	3113	11	22	3172	12	22	0.002
Anemia	13,655	11	1994	11	15	2951	11	22	2880	10	21	2873	10	21	2957	11	22	<0.001
COPD	12,179	10	1580	9	13	2503	9	21	2528	9	21	2659	9	22	2909	11	24	<0.001
Peripheral vascular diseases	6741	5	1012	6	15	1413	5	21	1418	5	21	1440	5	21	1458	5	22	0.002
Lipid metabolism disorders	6492	5	892	5	14	1384	5	21	1356	5	21	1450	5	22	1410	5	22	0.353
Dementia	3652	3	552	3	15	805	3	22	760	3	21	765	3	21	770	3	21	0.016
Liver diseases	3175	2	371	2	12	628	2	20	703	3	22	697	2	22	776	3	24	<0.001
Obesity	2800	2	337	2	12	528	2	19	574	2	21	666	2	24	695	3	25	<0.001

Lazio 2012–2021. Col, column; COPD, chronic obstructive pulmonary disease; DI, deprivation index.

The average follow-up was 4.25 years (SD, 3.18). In the very low DI group, the mean follow-up was 4.11 years (SD, 3.15), whereas in the very high DI group, it was 4.29 years (SD, 3.22) (*P* value < 0.001). During the period of the study (12 years), 57,158 patients died (45%), and 5994 (5%) developed ESKD that required KRTs. Table 2 reports the follow-up time and the CMR per 100 person-years and the crude ESKD rates per 100 person-years by DI. The overall CMR was 10.5 per 100 person-years. There were no substantial differences in CMR across DI classes. The overall ESKD crude rate was 1.11 per 100 person-years. There were no substantial differences in ESKD rates across DI classes.

**Table 2:** Patients with CKD, person-years of follow-up, number, percentage, and crude rate per 100 person-years of outcomes (mortality and ESKD) by DI

DI	*N*	Person-Years	Deaths		CMR	95% CI	ESKD		Crude ESKD Rate	95% CI
			*N*	%			*N*	%		
Total	127,457	542,192	57,158	44.8	10.5	10.5 to 10.6	5994	4.7	1.11	1.08 to 1.13
**DI**										
Very low	17,359	71,402	8103	46.7	11.3	11.1 to 11.6	820	4.7	1.15	1.07 to 1.23
Low	27,087	113,993	12,462	46.0	10.9	10.7 to 11.1	1174	4.3	1.03	0.97 to 1.09
Medium	27,799	119,889	12,189	43.8	10.2	10.0 to 10.3	1242	4.5	1.04	0.98 to 1.10
High	28,437	121,982	12,404	43.6	10.2	10.0 to 10.3	1385	4.9	1.14	1.08 to 1.20
Very high	26,775	114,926	12,000	44.8	10.4	10.3 to 10.6	1373	5.1	1.19	1.13 to 1.26

CI, confidence interval; CMR, crude mortality rate; DI, deprivation index.

**Figure 2:**
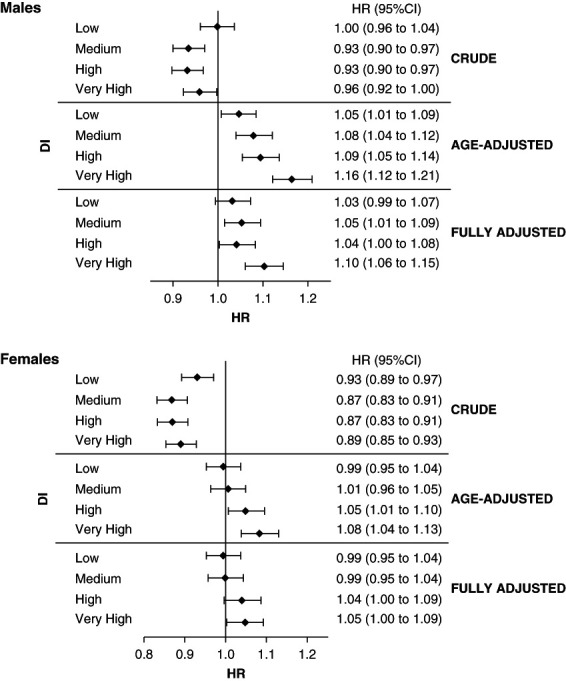
**Association between DI and mortality.** Crude, age-adjusted, and fully adjusted HR in male participants and female participants (reference class: very low DI). DI, deprivation index; HR, hazard ratio.

Figure 2 shows the association between DI and mortality. Age-adjusted models showed that men in the very high DI class were more likely to die than those in the very low DI class (HR, 1.16; 95% CI, 1.12 to 1.21). Among women, the corresponding values were HR, 1.08 (95% CI, 1.04 to 1.13). The other variables considered in the fully adjusted model slightly decreased the strength of the association. *P* values for interaction between sex and DI were 0.054, 0.020, and 0.045 in the crude, age-adjusted, and fully adjusted models, respectively.

**Figure 3:**
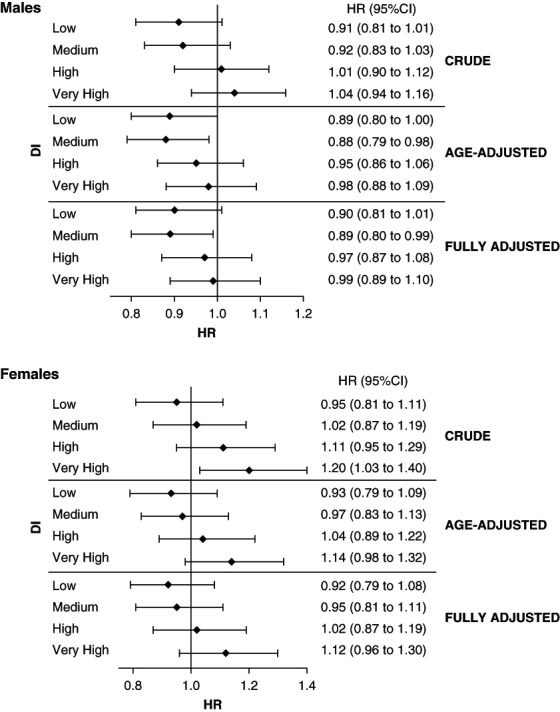
**Association between DI and ESKD.** Crude, age-adjusted, and fully adjusted HR in male participants and female participants (reference class: very low DI).

In the case of ESKD, sex was not an effect modifier of the association between the exposure and outcome. However, we decided to report the results of ESKD separately for men and women in the same way as those for mortality. Of 71,187 men, 3944 developed ESKD (6%). The competing risk model did not show a clear association between DI and ESKD. Of 56,270 women, 2050 developed ESKD (4%). There was no significant association between DI and the risk of developing ESKD. Adjusting for the other variables did not modify the results (Figure 3).

The graphical test to assess the proportional hazards assumption is shown in Supplemental Figure 1. The Schoenfeld residuals test confirmed the proportional hazards assumptions for both outcomes in both sexes (mortality *P* value = 0.599 in male and 0.081 in female, for ESKD *P* value = 0.388 in male and 0.630 in female).

## Discussion

This study analyzes the impact of social disadvantage on the outcomes associated with CKD in a large cohort with an extended follow-up. We found that patients with CKD belonging to the most deprived classes have a higher risk of death than least deprived patients. This association was stronger for men than for women. There was no evidence of association between SEP and the risk of progression to ESKD, and only men belonging to the medium DI class seemed to have a lower risk of developing ESKD.

Previous studies regarding the association of SEP and death in patients with CKD have been conducted in both Europe and the United States.^11,13,14^ In a study conducted in Scotland from 2010 to 2014, Solbu *et al.* showed that living in the most deprived areas was independently associated with mortality.^11^ SEP was obtained for 2950 patients using the Scottish Index of Multiple Deprivation. The cumulative HR (adjusted for sex, age, and eGFR) of the most deprived patients compared with the least deprived ones was 1.69 (95% CI, 1.19 to 2.40). This was higher than the HR obtained in our study, namely 1.16 (95% CI, 1.12 to 1.21) for men and 1.08 (95% CI, 1.04 to 1.13) for women, and could conceivably be explained by the inclusion of eGFR in the analytical model.^11^

In the United Kingdom, SEP has been measured using the score on the Index of Multiple Deprivation in a cohort of 918 patients^14^ based on education and occupational data from a questionnaire. The results from a multilinear regression model showed no significant association, probably because of the fact that the 3-year median follow-up in this cohort was too short to intercept all deaths.^14^

The largest cohort analyzed until now was in a study by Goldfarb-Rumyantzev *et al.* (13,400 patients from the Third National Health and Nutrition Examination Survey III).^13^ In this case, SEP was evaluated as the sum of multiple scores from a questionnaire. The results obtained using a multivariate regression model were expressed as HR, 2.70 (95% CI, 2.17 to 3.33), comparing the most deprived class with the least deprived, the latter HR being higher than ours. However, at least three aspects should be considered when comparing our results with those obtained by Goldfarb-Rumyantzev *et al.* First, the use of questionnaires to evaluate SEP could be biased by subjectivity. Second, the population was not representative of all American patients, but mainly of African Americans and Hispanic Americans. This aspect is essential because of the well-known effect of race on the severity of CKD. Finally, because the health care system in the United States is private, it is obvious that social deprivation has a greater effect on survival.

In accordance with most of the previous studies, we did not find any strong association between socioeconomic status and ESKD.^11,14,15^ In our cohort, the incidence of ESKD was similar to other studies. Still, given the high average age of our population, some cases of CKD could be interpreted as pathophysiological declines of GFR attributed to older age.^11,14^ Currently, the work by Weldegiorgis *et al.*^9^ is the only study to have found a higher risk of developing ESKD for participants in the most deprived categories compared with those in the least deprived classes. However, the results are not comparable with ours for the different study design, the diverse outcome definition, and the different area indicator of SEP used.

Interestingly, we found that patients in the mid-DI classes had a lower risk of developing ESKD than patients with very low DI. Our interpretation of these results is that ESKD is a very different outcome to death because it is also shaped by the availability of health care and individual health literacy. Indeed, patients with very high social deprivation could develop ESKD without being aware of it and, therefore, miss out on the possibility of treatment with KRTs. Another important aspect is that because the most deprived patients have a higher mortality rate, follow-ups could be interrupted before the occurrence of ESKD.

Socioeconomic inequalities in mortality are well described.^22^ In the Lazio region, several studies showed stronger inequalities in the incidence of a disease than in its outcomes, indicating that the major determinants of inequalities are individual lifestyle risk factors.^23–25^ Renal health has many determinants, both individual and social.^26^ It is clear that individual lifestyle factors play a considerable role in predicting the risk of developing CKD.^27^ Socioeconomic status is also believed to have a direct effect on several chronic conditions that, in turn, cause CKD.^7,8,28^ During CKD, the mechanisms that lead to death are intricate and only partially understood. Findings from the Chronic Renal Insufficiency Cohort Study reveal that adherence to healthy dietary patterns is associated with a lower risk for CKD progression and all-cause mortality.^29^ Because healthy diets are often expensive and less accessible to the most disadvantaged in society, these individuals could accumulate a higher risk of death during CKD due to newly discovered mechanisms.^30^

Health literacy is associated with the onset of CKD and all its clinical outcomes.^31,32^ Although we did not measure it directly, we think it contributes to the association between social deprivation and death and efforts should be made to promote it. Accordingly, the conclusions of the Kidney Disease Improving Global Outcomes Controversies Conference^33^ affirm that guidelines for primary care clinicians should be simplified to bridge the education gap.

SEP is a multifaceted aspect influenced by individual and collective characteristics, and finding a standardized measure for it has been a challenge for all researchers in this field. In the 1800s, Chadwick postulated that the place where a person lives can affect their health, hence the development of area-level measures. As previously mentioned, the DI is a composite indicator that considers many aspects of social conditions, and it guarantees a relatively faithful reproduction of actual social position.^21^ Notably, the score assigned to each patient is based on the census block, which includes 200 people and can therefore be used as an individual proxy.^21^ Another important consideration is that other studies produced in Europe have used similar indicators, making it possible to compare results from different studies.^11,14^

The main strength of our study is the reliability of our results because of the extended follow-up, the large cohort, which included the whole population of an Italian region, and the standardized measure of social deprivation. Despite this, our study also has some limitations that must be pointed out. First, we selected incident CKD cases by using an algorithm validated only for the prevalence CKD cases; the sensitivity was 51.0% with a specificity of 96.5%.^17,20^ These values are, however, still higher than those obtained by other European diagnostic codes.^6^ Applying a prevalence algorithm to identify incident cases could have slightly shortened the follow-up time because the real incident date is anterior to any possible identification through HIS. Moreover, we assume that this phenomenon affects all DI classes. It should be mentioned that despite being similar to indicators used in other countries to assess social deprivation, DI is based on the Italian census, thus being poorly generalizable to other settings.

Almost 12% of all incident patients with CKD were excluded because their DI information was missing because of the lack of coordinates of the residential address. These patients were located in rural areas to a greater extent than the individuals included in our cohort (44% versus 29%, respectively) and had a higher mortality (59% versus 45%, respectively). The geographical distribution of the DI is characterized by a high prevalence of low deprivation in metropolitan areas and high deprivation in rural areas. Therefore, our results would have been strengthened by the inclusion of these patients.

Importantly, the used algorithm does not include eGFR, and therefore, it does not provide any information about the CKD stage at the baseline, which could have affected the frequence of the ESKD events.

In conclusion, this study is, so far, the largest European cohort on social deprivation and CKD. The results support the importance for clinicians in considering the socioeconomic status in the screening process and the severity assessment of patients with CKD. Once CKD is diagnosed, our results underline the importance of strict monitoring of the most disadvantaged patients, who are at higher risk of death. Successive studies could analyze the mediating role of potential risk factors in the association between SEP and mortality to identify the mechanisms of this association, could include the development of strategies to address inequalities, and could use different SEP indicators to highlight the role of different SEP aspects. Furthermore, socioeconomic factors are key points for the health service governance, which must be guaranteed in an effective and fair manner for all citizens.

## Supplementary Material

## Data Availability

Data cannot be shared. The availability of the data are restricted for privacy reasons, and individual data are accessible following strict rules on the Lazio Region servers and cannot be exported. Aggregated data are available from the authors upon request (please contact Nera Agabiti, mail to: n.agabiti@deplazio.it).

## Supplemental Material

Supplemental Table 1. Codes used for the case identification.

Supplemental Table 2. Codes used for the identification of ESKD patients requiring KRTs.

Supplemental Table 3. Codes to identify baseline comorbidities.

Supplemental Figure 1. Log(−log[survival]) against log(time) for different levels of deprivation index. By outcomes and sex.
