# Supplementary material for: Socioeconomic Inequalities Worsen the Risk of Death in CKD: A Population-Based Cohort Study in Italy
Source: Kidney360. 2024 Sep 26;5(12):1853–61. doi: 10.34067/KID.0000000592 (PMC11687974; doi:10.34067/KID.0000000592)
Supplement: Supplementary file 1 [file kidney360-5-1853-s001.pdf]

ASN American Society of Nephrology | JASN Journal of the American Society of Nephrology | KIDNEY360 Accessing Our World From Every Angle | CJASN Clinical Journal of the American Society of Nephrology

ASN Journal Disclosure Form

As per ASN journal policy, I have disclosed any financial relationships or commitments I have held in the past 36 months as included below. I have listed my Current Employer below to indicate there is a relationship requiring disclosure. If no relationship exists, my Current Employer is not listed.

N. Agabiti reports the following:
Employer: Department of Epidemiology

I understand that the information above will be published within the journal article, if accepted, and that failure to comply and/or to accurately and completely report the potential financial conflicts of interest could lead to the following: 1) Prior to publication, article rejection, or 2) Post-publication, sanctions ranging from, but not limited to, issuing a correction, reporting the inaccurate information to the authors' institution, banning authors from submitting work to ASN journals for varying lengths of time, and/or retraction of the published work.

Name: Nera Agabiti
Manuscript ID: K360-2024-000260R1
Manuscript Title: Socioeconomic Inequalities Worsen the Risk of Death in Chronic Kidney Disease: A Population-Based Cohort Study in Italy.
Date of Completion: July 24, 2024
Disclosure Updated Date: July 24, 2024

ASN American Society of Nephrology · JASN Journal of the American Society of Nephrology · KIDNEY360 Accessing Our World From Every Angle · CJASN Clinical Journal of the American Society of Nephrology

ASN Journal Disclosure Form

As per ASN journal policy, I have disclosed any financial relationships or commitments I have held in the past 36 months as included below. I have listed my Current Employer below to indicate there is a relationship requiring disclosure. If no relationship exists, my Current Employer is not listed.

G. Cesaroni reports the following:
Employer: ASL Roma 1

I understand that the information above will be published within the journal article, if accepted, and that failure to comply and/or to accurately and completely report the potential financial conflicts of interest could lead to the following: 1) Prior to publication, article rejection, or 2) Post-publication, sanctions ranging from, but not limited to, issuing a correction, reporting the inaccurate information to the authors' institution, banning authors from submitting work to ASN journals for varying lengths of time, and/or retraction of the published work.

Name: Giulia Cesaroni
Manuscript ID: K360-2024-000260R2
Manuscript Title: Socioeconomic Inequalities Worsen the Risk of Death in Chronic Kidney Disease: A Population-Based Cohort Study in Italy
Date of Completion: September 3, 2024
Disclosure Updated Date: September 3, 2024

ASN
American Society of Nephrology
JASN
Journal of the American Society of Nephrology
KIDNEY360
Accessing Our World From Every Angle
CJASN
Clinical Journal of the American Society of Nephrology

ASN Journal Disclosure Form

As per ASN journal policy, I have disclosed any financial relationships or commitments I have held in the past 36 months as included below. I have listed my Current Employer below to indicate there is a relationship requiring disclosure. If no relationship exists, my Current Employer is not listed.

M. Davoli reports the following:
Employer: Department of Epidemiology - Lazio Regional Health Service

I understand that the information above will be published within the journal article, if accepted, and that failure to comply and/or to accurately and completely report the potential financial conflicts of interest could lead to the following: 1) Prior to publication, article rejection, or 2) Post-publication, sanctions ranging from, but not limited to, issuing a correction, reporting the inaccurate information to the authors' institution, banning authors from submitting work to ASN journals for varying lengths of time, and/or retraction of the published work.

Name: Marina Davoli
Manuscript ID: K360-2024-000260R1
Manuscript Title: Socioeconomic Inequalities Worsen the Risk of Death in Chronic Kidney Disease: A Population-Based Cohort Study in Italy
Date of Completion: July 23, 2024
Disclosure Updated Date: July 23, 2024

ASN
American Society of Nephrology
JASN
Journal of the American Society of Nephrology
KIDNEY360
Accessing Our World From Every Angle
CJASN
Clinical Journal of the American Society of Nephrology

ASN Journal Disclosure Form

As per ASN journal policy, I have disclosed any financial relationships or commitments I have held in the past 36 months as included below. I have listed my Current Employer below to indicate there is a relationship requiring disclosure. If no relationship exists, my Current Employer is not listed.

P. Ferraro reports the following:
Employer: Università degli Studi di Verona; Consultancy: Alnylam, AstraZeneca, Bayer, Novo Nordisk; Research Funding: Alnylam; Honoraria: AstraZeneca, Gilead, Alnylam; Patents or Royalties: UpToDate; Advisory or Leadership Role: Editorial Board of Nutrients, Journal of Nephrology, Kidney and Blood Pressure Research; Board of ERKReg; Board of ERA Registry; Secretary of the Italian Registry of Dialysis and Transplantation; and Speakers Bureau: AstraZeneca, Gilead, Alnylam.

I understand that the information above will be published within the journal article, if accepted, and that failure to comply and/or to accurately and completely report the potential financial conflicts of interest could lead to the following: 1) Prior to publication, article rejection, or 2) Post-publication, sanctions ranging from, but not limited to, issuing a correction, reporting the inaccurate information to the authors' institution, banning authors from submitting work to ASN journals for varying lengths of time, and/or retraction of the published work.

Name: Pietro Manuel Ferraro
Manuscript ID: K360-2024-000260R2
Manuscript Title: Socioeconomic Inequalities Worsen the Risk of Death in Chronic Kidney Disease: A Population-Based Cohort Study in Italy
Date of Completion: September 14, 2024
Disclosure Updated Date: March 26, 2024

ASN
American Society of Nephrology
JASN
Journal of the American Society of Nephrology
KIDNEY360
Accessing Our World From Every Angle
CJASN
Clinical Journal of the American Society of Nephrology

ASN Journal Disclosure Form

As per ASN journal policy, I have disclosed any financial relationships or commitments I have held in the past 36 months as included below. I have listed my Current Employer below to indicate there is a relationship requiring disclosure. If no relationship exists, my Current Employer is not listed.

M. Giaccari has nothing to disclose.

I understand that the information above will be published within the journal article, if accepted, and that failure to comply and/or to accurately and completely report the potential financial conflicts of interest could lead to the following: 1) Prior to publication, article rejection, or 2) Post-publication, sanctions ranging from, but not limited to, issuing a correction, reporting the inaccurate information to the authors' institution, banning authors from submitting work to ASN journals for varying lengths of time, and/or retraction of the published work.

Name: Marta Giaccari
Manuscript ID: K360-2024-000260R1
Manuscript Title: Socioeconomic Inequalities Worsen the Risk of Death in Chronic Kidney Disease: A Population-Based Cohort Study in Italy
Date of Completion: July 19, 2024
Disclosure Updated Date: July 19, 2024

ASN
American Society of Nephrology
JASN
Journal of the American Society of Nephrology
KIDNEY360
Accessing Our World From Every Angle
CJASN
Clinical Journal of the American Society of Nephrology

ASN Journal Disclosure Form

As per ASN journal policy, I have disclosed any financial relationships or commitments I have held in the past 36 months as included below. I have listed my Current Employer below to indicate there is a relationship requiring disclosure. If no relationship exists, my Current Employer is not listed.

C. Marino reports the following:
Employer: Department of Epidemiology of the Regional Health Service, ASL Roma 1

I understand that the information above will be published within the journal article, if accepted, and that failure to comply and/or to accurately and completely report the potential financial conflicts of interest could lead to the following: 1) Prior to publication, article rejection, or 2) Post-publication, sanctions ranging from, but not limited to, issuing a correction, reporting the inaccurate information to the authors' institution, banning authors from submitting work to ASN journals for varying lengths of time, and/or retraction of the published work.

Name: Claudia Marino
Manuscript ID: K360-2024-000260R1
Manuscript Title: Socioeconomic Inequalities Worsen the Risk of Death in Chronic Kidney Disease: A Population-Based Cohort Study in Italy.
Date of Completion: July 19, 2024
Disclosure Updated Date: July 19, 2024
