# Supplementary material for: Socioeconomic Inequalities Worsen the Risk of Death in CKD: A Population-Based Cohort Study in Italy
Source: Kidney360. 2024 Sep 26;5(12):1853–61. doi: 10.34067/KID.0000000592 (PMC11687974; doi:10.34067/KID.0000000592)

Supplementary Table 1. Codes used for the case identification.

Name	Code
**Hospital Discharge Registry**	**ICD-9-CM**
Diagnoses	
Diabetes with renal manifestations	250.4X
Hypertensive chronic kidney disease	403.XX
Hypertensive heart and chronic kidney disease	404.XX
Chronic glomerulonephritis	582.XX
Nephritis and nephropathy not specified as acute or chronic	583.XX
Chronic kidney disease (ckd)	585.XX
Renal failure, unspecified	586.XX
Renal sclerosis, unspecified	587.XX
Disorders resulting from impaired renal function	588.XX
Cystic kidney disease	753.1X
Chronic pyelonephritis	590.0X
Encounter for dialysis and dialysis catheter care	V56.X
Kidney replaced by transplant	V42.0
Procedures	
Hemodialysis	39.95
Peritoneal dialysis	54.98
Transplant of kidney	55.6X
Arteriovenostomy for renal dialysis	39.27
Creation of cutaneoperitoneal fistula	54.93
Revision of arteriovenous shunt for renal dialysis	39.42
Removal of arteriovenous shunt for renal dialysis	39.43
Venous catheterization for renal dialysis	38.95
Closed [percutaneous] [needle] biopsy of kidney	55.23
**Outpatient Specialist Service Information System**	**Regional Code**
Complex outpatient services for	
Assess diagnosis of nephropathies	P583
Assess chronic kidney disease	P585A
Assess kidney transplant	P585B
Follow-up of kidney transplant patient	PV420
Dialytic Treatments	V56.X
Other services	
First ambulatory specialist visit (nephrology)	89.7(29)
Ambulatory specialist visit (nephrology)	89.01(29)
Measurement of urine albumin	90.33.4
Definition of the haemodialysis or peritoneal dialysis scheme (nephrology)	89.03(29)
Hemodialysis or hemodiafiltration	39.95.X
Peritoneal dialysis	54.98.X
Venous catheterization for renal dialysis	38.95
Creation of cutaneoperitoneal fistula (peritoneal catheter)	54.93
Debriding of peritoneal catheter	39.99.1
Removal of peritoneal catheter	97.82
Revision of peritoneal catheter	97.29.1
**Copayment Exemption Registry**	**Regional Code**
Chronic Kidney disease	052.585
Kidney transplantation	052.V42.0
**Drug Dispensing Registry**	**ATC**
Erythropoietin	B03XA01
Darbepoetin alfa	B03XA02
Methoxy polyethylene glycol-epoetin beta	B03XA03
Polystyrene sulfonate	V03AE01
Sevelamer	V03AE02
Lanthanum carbonate	V03AE03
Sucroferric oxyhydroxide	V03AE05

Supplementary Table 2. Codes used for the identification of ESKD patients requiring kidney replacement therapies.

Name	Code
**Hospital Discharge Registry**	**ICD-IX-CM**
Diagnoses	
Chronic kidney disease (ckd)	585.6
Encounter for dialysis and dialysis catheter care	V56.X
Kidney replaced by transplant	V42.0
Dialytic Treatments	V56.X
Procedures	
Hemodialysis	39.95
Peritoneal dialysis	54.98
Transplant of kidney	55.6X
Arteriovenostomy for renal dialysis	39.27
Creation of cutaneoperitoneal fistula	54.93
Revision of arteriovenous shunt for renal dialysis	39.42
Removal of arteriovenous shunt for renal dialysis	39.43
Venous catheterization for renal dialysis	38.95
**Outpatient Specialist Service Information System**	**Regional Code**
Complex outpatient services for	
Follow-up of kidney transplant patient	PV420
Other services	
Definition of the haemodialysis or peritoneal dialysis scheme (nephrology)	89.03 (29)
Hemodialysis or hemodiafiltration	39.95.X
Peritoneal dialysis	54.98.X
Venous catheterization for renal dialysis	38.95
Creation of cutaneoperitoneal fistula (peritoneal catheter)	54.93
Debriding of peritoneal catheter	39.99.1
Removal of peritoneal catheter	97.82
Revision of peritoneal catheter	97.29.1
**Copayment Exemption Registry**	
Kidney transplantation	052.V42.0

Supplementary Table 3. Codes to identify baseline comorbidities.

Condition	ICD-9-CM codes
Cancer	140-208, V10
Diabetes	250
Obesity	270.0
Lipid metabolism disorders	272
Anemia	280-284, 285 (excl. 285.1)
Dementia	290.0-290.4, 294.1, 331.0
Hypertension	401-405
Ischemic cardiopathy	410-414, 429.7
Arrhythmias	426, 427
Heart failure	428
Cerebrovascular disease	430-438
Peripheral vascular disease	440-448, 557
Chronic obstructive pulmonary disease	490-496
Liver disease	571-573

Supplementary Figure 1. Log(-log(survival)) against log(time) for different levels of Deprivation Index. By outcomes and sex.

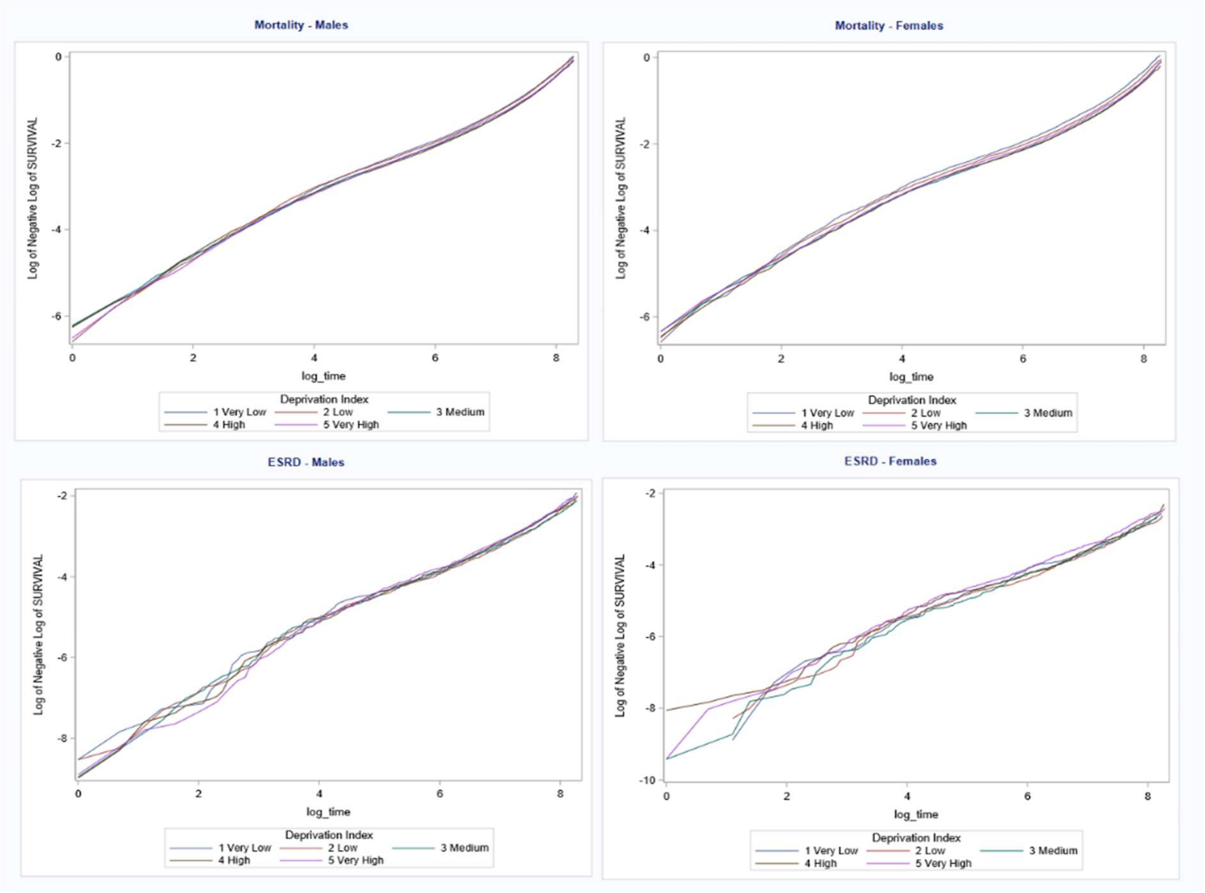

Supplement: Supplementary file 2 [file kidney360-5-1853-s002.pdf]
